# Unlocking natural history collections to improve eDNA reference databases and biodiversity monitoring

**DOI:** 10.1093/biosci/biaf140

**Published:** 2025-09-11

**Authors:** Sarah Schmid, Nicolas Straube, Camille Albouy, Bo Delling, James Maclaine, Michael Matschiner, Peter Rask Møller, Annamaria Nocita, Anja Palandačić, Lukas Rüber, Moritz Sonnewald, Nadir Alvarez, Stéphanie Manel, Loïc Pellissier

**Affiliations:** Ecosystems and Landscape Evolution group in between the Swiss Federal Institute for Forest, Snow and Landscape Research, Birmensdorf, Switzerland; ETH Zürich, Zürich, Switzerland; University Museum of Bergen, Bergen, Norway; Ecosystems and Landscape Evolution group in between the Swiss Federal Institute for Forest, Snow and Landscape Research, Birmensdorf, Switzerland; ETH Zürich, Zürich, Switzerland; Swedish Museum of Natural History, Stockholm, Sweden; Natural History Museum, London, England, United Kingdom; Bavarian State Collection of Zoology, Munich, Germany; Ludwig Maximilians University Munich, Munich Planneg, Germany; Natural History Museum, University of Oslo, Oslo, Norway; Natural History Museum of Denmark, Copenhagen, Denmark; Museum La Specola, Florence, Italy; Museum of Natural History Vienna, Vienna, Austria; Department of Biology, University of Ljubljana, Ljubljana, Slovenia; Natural History Museum of Bern, Bern, Switzerland; Institute of Ecology and Evolution, University of Bern, Bern, Switzerland; Senckenberg Research Institute, Frankfurt am Main, Germany; Naturéum—State Museum of Natural History of the Canton de Vaud, Lausanne, Switzerland; Department of Ecology and Evolution, University of Lausanne, Lausanne, Switzerland; Ecole Pratique des Hautes Etudes and is based, Centre d'Ecologie Fonctionnelle et Evolutive, Montpellier, France; Institut Universitaire de France, Paris, France; Ecosystems and Landscape Evolution group in between the Swiss Federal Institute for Forest, Snow and Landscape Research, Birmensdorf, Switzerland; ETH Zürich, Zürich, Switzerland

**Keywords:** museomics, fish, metabarcoding, historical DNA, museum specimens

## Abstract

Biodiversity changes due to human activities highlight the need for efficient biodiversity monitoring approaches. Environmental DNA (eDNA) metabarcoding offers a noninvasive method used for biodiversity monitoring and ecosystem assessment, but its accuracy depends on comprehensive DNA reference databases. Natural history collections often contain rare or difficult-to-obtain samples that can serve as a valuable resource to fill gaps in eDNA reference databases. In the present article, we discuss the utility of specimens from natural history collections in supporting future eDNA applications. Museomics—the application of -omics techniques to museum specimens—offers a promising avenue for improving eDNA reference databases by increasing species coverage. Furthermore, museomics can provide transferable methodological advancements for extracting genetic material from samples with low and degraded DNA. The integration of natural history collections, museomics, and eDNA approaches has the potential to significantly improve our understanding of global biodiversity, highlighting the continued importance of natural history collections.

We are witnessing a significant change in Earth's biological diversity driven by anthropogenic factors (Pereira et al. 2010), resulting in the geographic redistribution of species (Lenoir et al. 2020, Chevalier et al. 2024) and the spatiotemporal reconfiguration of communities in both terrestrial and aquatic biomes (Walsh et al. 2015, García-Navas et al. 2020). Considering the speed of the environmental modifications induced by global changes, it is crucial to assess the shift in the distribution of various taxonomic groups and to pinpoint areas where species are most at risk to enable effective planning and resource allocation. Traditional monitoring survey methods often miss elusive or rare species (Boussarie et al. 2018, Mathon et al. 2022), suffer from a geographic bias in sampling efforts, and are ill suited to detect rapid modifications of species community composition induced by climate change (Staudinger et al. 2013). Environmental DNA (eDNA) metabarcoding has been proposed to gather species occurrences for various taxa and ecosystems more efficiently (Valentini et al. 2016, Pereira et al. 2021) and to facilitate the monitoring of anthropogenic impact on biodiversity dynamics.

eDNA metabarcoding has the potential to speed up the collection of species distribution information by analyzing genetic material obtained from environmental samples (eDNA) that contain a mixture of intra- and extracellular DNA, without the need to collect individuals from the ecosystem (Taberlet et al. 2012). The process involves species detection through water or air filtration (Clare et al. 2022), soil sampling (Allen et al. 2023), or surface swabbing (Aucone et al. 2023), followed by amplification and sequencing of one or more DNA barcodes (see below), which are then compared with a genetic reference database for species identification (Fraija-Fernández et al. 2020). This noninvasive approach has been applied to detect a range of organisms, including fishes (Ramírez-Amaro et al. 2022, Rozanski et al. 2022), and has demonstrated the ability to detect species occurrences of elusive species such as sharks (Bakker et al. 2017, Boussarie et al. 2018), large pelagic species (Veron et al. 2023), and cryptic species like gobies (Boulanger et al. 2021). For eDNA metabarcoding to be an effective tool for conservation, accurate assignment of eDNA sequences to the correct species is essential. Species-level detection is critical for various applications of eDNA metabarcoding, ranging from informing management decisions to detecting invasive species (Ruppert et al. 2019). This process depends on comprehensive DNA reference databases (Keck et al. 2022).

DNA reference databases generally consist of short DNA sequences called barcodes (usually between 100 to 700 base pairs) that are taxonomically annotated and curated. A fragment of the cytochrome c oxidase I gene (COI) of 650 base pairs is generally considered the universal DNA barcode for metazoans (Hebert et al. 2003). Although COI is widely used for assessing animal diversity in environmental samples, it offers lower resolution and overall performance in assigning a sequence to a species compared with other barcodes in some taxa such as fish (Collins et al. 2019). In consequence, alternative barcodes have been developed to improve species specificity during PCR (polymerase chain reaction) amplification. For example, primers targeting the ribosomal-RNA 16S and 12S genes provide higher resolution and better specificity for specific taxa such as fish (e.g., Miya et al. 2015, Valentini et al. 2016). Because eDNA studies rely on PCR amplification of specific markers, reference databases should be constructed to align with the amplicons typically used in these workflows to enable accurate species (or taxa) identification. Although it is possible to monitor biodiversity using molecular operational taxonomic units without linking sequences to identified species (Marques et al. 2020), assigning barcodes at the species level improves the detection of threatened, rare, endemic, or invasive species, enhancing the ecological and conservation relevance of eDNA data (Beng and Corlett 2020).

Current public databases such as GenBank, RefSeq, and the Barcode of Life Data System offer valuable resources (Leray et al. 2019). However, they also present limitations, including inconsistent link to verified taxonomic vouchers (Locatelli et al. 2020), potential sequence errors (Meiklejohn et al. 2019), and marker biases, particularly towards COI (figure 1; Ratnasingham and Hebert 2007). Although reference databases are central to assigning eDNA sequences to taxa, their coverage remains incomplete across different taxonomic groups and marker types (figure 1; Marques et al. 2021). For example, COI sequences are available for over 25,000 fish species, surpassing other barcodes such as 12S or 16S, which, despite being frequently used in eDNA metabarcoding, lack comprehensive taxonomic coverage (figure 1a). Newer genomic resources, including complete mitochondrial genomes and raw genomic data from the Sequence Read Archive, are growing rapidly (figure 1a) but are not yet systematically integrated into curated reference databases (but see Medeiros et al. 2025). This discrepancy between marker usage in eDNA studies and available reference sequences limits the accuracy of species identification and ultimately hampers the wider use of eDNA in conservation (Beng and Corlett 2020).

**Figure 1.**
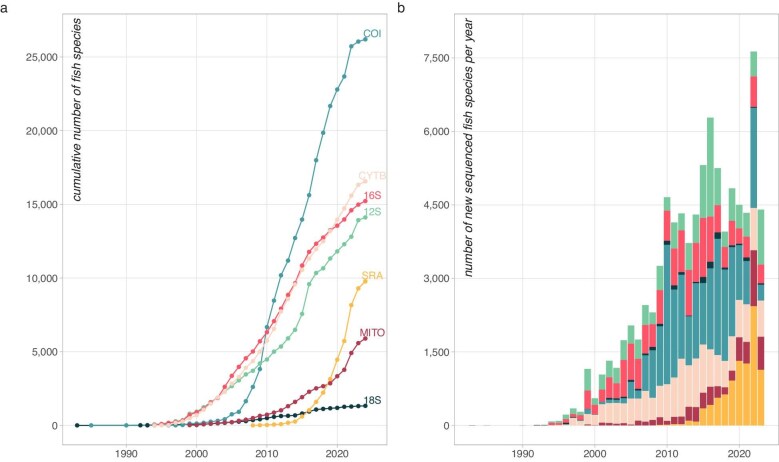
Cumulative number of species sequenced for a given barcode or type of genomic data. (a) The lines correspond to the cumulative number of fish species with a given barcode or genomic data available. The values were retrieved from NCBI for all fish species (marine and freshwater). (b) The bar plots correspond to the number of new species added each year for a given barcode or genomic data. Abbreviations: 12S, 12S ribosomal RNA (light green); 16S, 16S ribosomal RNA (pink); 18S, 18S ribosomal RNA (dark blue); COI, cytochrome oxidase I (blue); CYTB, cytochrome B (light pink); MITO, complete mitochondrial genome (dark red); SRA, Sequence Read Archive, which consists of various types of genomic data (yellow).

To address these gaps, scientific consortia and working groups are currently working globally to create and maintain high-quality reference databases (e.g., MIDORI2, PFR2, dinoref, PhytoREF, Mare-MAGE). In addition, an increasing number of projects aim at sequencing the entire genomes of specific target species sets (e.g., the Global Invertebrate Genomics Alliance, the Earth BioGenome Project, the European Reference Genome Atlas, the Vertebrate Genomes Project; see Formenti et al. 2022 for references). Designed primarily to deliver high-quality whole-genome assemblies, these initiatives seek to offer novel perspectives on genomic diversity and architecture. They also advance metagenomic eDNA studies by expanding comprehensive reference data and enrich metabarcoding research by supplying a broader pool of barcodes regions. Although samples for such projects can be obtained from biobanks, *in situ* collection of individuals is more frequent, which is not only time consuming and costly, but also requires taxonomic expertise to achieve accurate species identification. Furthermore, species missing from databases are often rare and elusive, making the sampling of living organisms practically, financially, and ethically challenging. An alternative is to leverage the wealth of scientific collection material preserved in natural history museum collections as a source of genetic information for underrepresented taxa.

## Natural history museum collections in support of eDNA-based projects

Biological natural history museum collections consist of a vast number of specimens that can be grouped into two broad categories: dry and wet collections. Dry collections are composed of samples that have been dried rather than being stored in a liquid preservation solution such as formalin or ethanol. These collections can include specimens such as pinned insects, stuffed fish, bones, or pressed plants. Wet collections, in contrast, consist of specimens that might have been fixed with formalin and subsequently stored in a preservative liquid such as 75% ethanol. Wet specimens can include a variety of organisms, from small invertebrates to larger animals such as fish or reptiles, as well as organ or tissue samples. Natural history museum collections also house type specimens—the individual specimens on which species descriptions are based. They serve as authoritative references and, by definition, bear the correct names, even when taxonomic perspectives shift and species are moved to different genera or assigned new rank (Renner et al. 2024). Therefore, type specimens are crucial resources to solve species synonymy and to identify species complexes (Sluys 2021), in addition to sometimes constituting the sole known record for a taxon (e.g., Kirchman et al. 2010).

Natural history museum collections are therefore exceptional repositories of taxonomic knowledge (Winker 2004), genetic source material (Wandeler et al. 2007), and historical and ecological data (Fong et al. 2023, Jones et al. 2024). They can enhance our understanding of species distribution over time (Elith et al. 2006, Baer et al. 2023) or morphological adaptation to climate change (MacLean et al. 2018), and they can inform the assessment of a species’ conservation status (Mollen and Iglésias 2023). Their contributions extend beyond natural sciences research, in fields such as public health (Cook et al. 2020) and education (Ellwood et al. 2020, Leerhøi et al. 2024). The long-term value of these collections to society emphasizes the need for their preservation over time, as they may hold unforeseen benefits (Miller et al. 2020) even as their funding decreases (Bradley et al. 2014). Indeed, numerous collection-based studies, especially those involving genetic analyses, are built on samples that were initially gathered for a different purpose. With technical advancements, they can reach new audiences and provide answers to novel scientific questions (Meineke et al. 2018, Heberling et al. 2019, Lauridsen et al. 2022, Davis and Knapp 2024).

### The value of natural history museum collections for eDNA barcode reference databases

**Figure 2.**
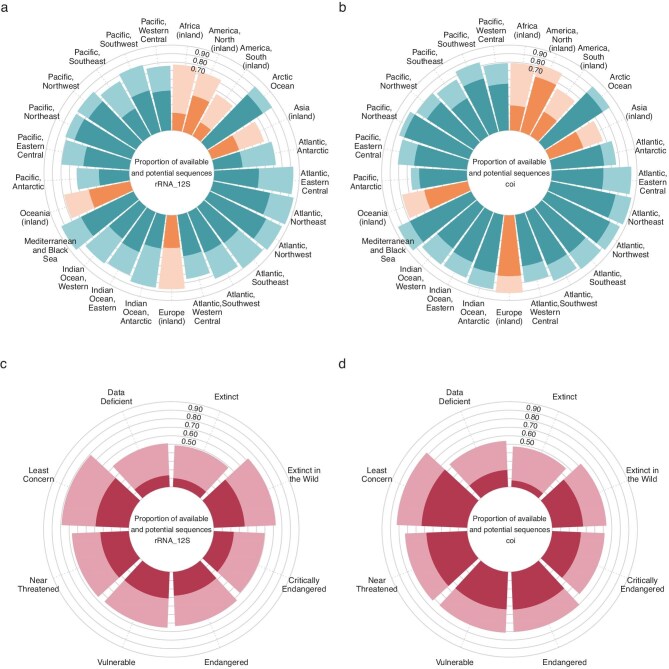
The potential of fish museum specimens to improve the reference database for two highly used mitochondrial genes in eDNA studies (12S ribosomal RNA [12S] and cytochrome C oxidase I [COI]; other genes are displayed in supplemental figures S1 and S2). The proportion of sequences available is highlighted in the dark color. Potential new sequences based on available museum specimens in European collections are displayed in the lighter color. (a) The potential of improvement for 12S gene according to geographic regions. The regions in blue are hosting marine fish species, and the regions in orange are for freshwater species (inland regions). (b) The potential of improvement for COI gene according to geographic regions. The regions in blue are hosting marine species, and the regions in orange are for freshwater species (inland regions). (c) The potential of improvement for 12S gene according to global IUCN Red List of Threatened Species assessments for marine and freshwater fishes. (d) The potential of improvement for COI gene according to global IUCN Red List of Threatened Species assessments for marine and freshwater fishes. The complete list of fish species was retrieved from FishBase using the R package rfishbase (Boettiger et al. 2012), as well as their corresponding geographic distribution. The museum data were retrieved from Global Biodiversity Information Facility (GBIF) datasets (Chagnoux et al. 2024, Delling et al. 2024, Dondorp and Creuwels 2024, Frafjord et al. 2023, Herder 2025, Lenuk et al. 2024, Møller and Carl 2025, Museum für Naturkunde Berlin 2018, National Museum in Prague 2014, Natural History Museum 2024, Pauwels et al. 2025, Quesada and Villaronga 2025, Senckenberg 2022a, 2022b, Solís Fraile 2020, Straube 2024, University of Oslo 2021). The IUCN Red List assessments were retrieved from the IUCN website.

Leveraging natural history museum collections in support of eDNA-based projects exemplifies the constantly evolving usage of museum specimens. Using fish as a case study, we show that these collections hold considerable potential to improve reference databases for commonly used eDNA barcodes, such as 12S and COI (figure 2; see supplemental figures S1 and S2 for additional barcodes). In many cases, the number of species represented in museum collections exceeds those currently covered in public databases. This underscores the value of existing material to close taxonomic and geographic gaps in reference databases. In addition to greatly improving database coverage for continents such as Africa and, notably, for freshwater species (figure 2a, 2b), natural history museum collections also host numerous specimens of species listed as Threatened on the IUCN Red List (figure 2c, 2d; IUCN 2024). These specimens might be the only available source of genetic information for those rare, elusive, or extinct species, which are practically and ethically difficult to sample in the wild.

Furthermore, museum specimens serve as authoritative vouchers, enabling the direct link of DNA sequences to the original specimen, which is especially valuable when using type specimens (see above). This ensures reproducibility in genetic studies and prevents the propagation of taxonomic errors (Buckner et al. 2021). Finally, natural history museum collections frequently contain multiple specimens of a given species sampled across its geographic distribution. This diversity of specimens is valuable for eDNA databases, as it allows capturing the intraspecific genetic variability of species. By incorporating multiple sequences per taxon (e.g., from different geographic locations), the eDNA reference database can be enhanced to more comprehensively represent the target species, ensuring more accurate species identification (Blackman et al. 2023).

## Barcoding of historical museum specimens for eDNA reference databases

Recently collected voucher specimens have been commonly used to generate barcode sequences for eDNA reference databases using conventional amplicon-based workflows (de Santana et al. 2021, Bemis et al. 2023). These samples are often stored in ethanol or frozen, and contain high-quality DNA; they are consequently compatible with standard PCR and sequencing protocols. However, they differ from historical museum specimens, which often require specific strategies owing to DNA degradation.

Sanger sequencing was initially used for barcoding museum specimens but has limited throughput and sensitivity for degraded DNA (Strutzenberger et al. 2012, Hebert et al. 2013). Next-generation sequencing has since become the standard approach, enabling high-throughput recovery of barcode sequences even from degraded material. Next-generation sequencing enabled high-throughput amplicon sequencing of historical specimens on platforms such as 454 pyrosequencing (Xu et al. 2015); Illumina MiSeq (Forin et al. 2018, Dopheide et al. 2022); and PacBio, which uses single molecule real-time sequencing (D'Ercole et al. 2021, Levesque-Beaudin et al. 2023).

To prepare amplicon products for next-generation sequencing, a two-step PCR is typically required. This approach involves an initial PCR to amplify the barcode of interest, followed by a second PCR using tailed primers containing a multiple identifier, enabling the pooling of multiple samples into a single sequencing reaction. This method has recovered full-length barcode sequences from century-old dried museum material (Forin et al. 2018, Haran et al. 2018). However, historical specimens—for instance, those from wet collections having experienced formaldehyde fixation—often suffer from severe DNA fragmentation, base damage, chemical alterations such as cross-links, and contamination (Raxworthy and Smith 2021; see box 1). In such cases, fragments longer than 200 base pairs may be rare and primer binding sites truncated (Allentoft et al. 2012), making full-length barcode recovery difficult.

Box 1. **Addressing the challenges of using museum specimens in molecular biology research.**

Modern natural history collections include both historic material and recent tissues, which have the potential to enhance genetic reference databases for eDNA metabarcoding. Most specimens are historic and were not curated with preserving DNA integrity in mind. Their curatorial treatment frequently involves fixation with formaldehyde, which is efficient for preserving morphological characters, but damages DNA (Simmons 2014). It causes cross-linking, hydrolysis and methylol adducts, which inhibit molecular techniques required to access the DNA sequence (e.g., Ruane and Austin 2017). Furthermore, handling histories are often undocumented, making it difficult to determine preservation methods. For instance, it is often unclear whether the specimens were preserved in ethanol or formaldehyde, and for what duration, leading to high variability in DNA quality (Cook et al. 2014, McDonough et al. 2018). Consequently, wet collection specimens—unlike dry collections, which generally yield higher sequencing success—remain challenging to process, and require optimized protocols for extraction and library preparation (Raxworthy and Smith 2021).

**DNA extraction**

Formaldehyde-related DNA damage was initially addressed in the medical field, where histological samples are routinely fixed with formaldehyde and commercial kits were developed to tackle cross-linking (e.g., QIAamp DNA FFPE Tissue Kit, QIAGEN; EchoLUTION FFPE DNA Kit, BioEcho). However, museum specimens may have been exposed to higher concentrations and longer fixation times, exacerbating DNA degradation. As a result, custom protocols tailored for museum specimens were developed to recover small DNA fragments and disrupt cross-links (Straube et al. 2021, Hahn et al. 2022). These protocols are often time consuming, making commercial kits appealing. Although no commercial DNA extraction kit exists specifically for museum material to our knowledge, FFPE (for *formalin-fixed paraffin-embedded* tissue) and ancient DNA kits are possible alternatives.

**DNA library preparation**

Library preparation also requires tailored protocols. The degraded nature of DNA of museum specimens necessitates modifications of standard library preparation protocols. This challenge has been extensively addressed in ancient DNA studies (Gansauge et al. 2020, Kapp et al. 2021). One major advance is the development of single-stranded DNA (ssDNA) library preparation, which captures damaged and short molecules often missed by double-stranded methods (Wales et al. 2015). Although more time consuming and costly, ssDNA protocols have been critical in ancient and historical DNA studies (Dabney et al. 2013, Gansauge and Meyer 2013), inspiring recent protocols specific to museum samples (Straube et al. 2021) and now available as commercial kits (xGen ssDNA and Low-Input DNA Library Prep Kit, IDT; SRSLY NGS Library Prep Kit, ClaretBio). Libraries can be directly sequenced or enriched via hybrid-capture protocols (Lemmon and Lemmon 2013).

**Custom protocols or kits? Tailoring lab workflow to project scale and resources**

The choice between custom protocols and commercial kits depends on project needs. Commercial kits require less technical expertise through ready-to-use reagents, whereas custom protocols require solution preparation, increasing hands-on time and risk of contamination. However, custom protocols often have lower per-sample costs, despite higher initial financial investment to purchase stock reagents (respectively €42.90 and €31, excluding sequencing on the basis of the combination of the *EchoLUTION FFPE DNA Kit* and *SRSLY PicoPlus Uracil+NGS library Prep Kit* versus Straube et al. 2021 protocol). Working time costs for custom protocols, however, may still be higher due increased hand-on times. Therefore, commercial kits may be best for short-term projects with limited samples, whereas custom protocols may be more cost effective and adaptable for long-term or large-scale projects.

Nested PCR is an effective approach to address such issues. It uses multiple rounds of amplification to target overlapping short fragments of a barcode region, eventually leading to the reconstruction of the complete barcode. For example, two-stage nested multiplex PCR has been used to reconstruct full-length barcodes from historical specimens by amplifying 100–150 base-pair regions with overlapping primers (Mitchell 2015, Prosser et al. 2016). Likewise, a three-step nested PCR approach, involving an initial PCR targeting near full-length loci followed by a second PCR using nested primers to amplify smaller fragments, facilitated barcode recovery in degraded ethanol-preserved insects (Akankunda et al. 2020).

An alternative strategy is the use of mini-barcodes, which can provide a level of information similar to that provided by full-length barcodes (Hajibabaei et al. 2006, Yeo et al. 2020). Mini-barcodes have been used successfully in various studies based on historical specimens, from birds (Patel et al. 2010) to arthropods (Shokralla et al. 2011, Velasco-Cuervo et al. 2019). The reduced size of mini-barcodes makes them more likely to be retrieved, even in samples with degraded DNA, and computational methods are available to identify the most informative mini barcode regions for a given gene and taxon (Boyer et al. 2012).

Despite these advances, the success of amplicon-based methods still decreases with the specimen’s age (Hebert et al. 2013, Xu et al. 2015). This has motivated the development of primer-free strategies, such as capture-based protocols, which use short, synthesized oligonucleotide probes to hybridize with target barcode regions. These methods can enrich and recover sequences even when primer binding sites are damaged (Agne et al. 2022a, Landry et al. 2023). This approach, sometimes referred to as *barcode*  *fishing* (Rancilhac et al. 2020), has been successfully applied to recover barcodes of wet-collection type specimens (e.g., Agne et al. 2022b, Scherz et al. 2020), demonstrating its utility for highly degraded and historically valuable material.

Although these methods have enabled the extension of DNA barcoding to historical specimens to improve eDNA reference databases, the effort and cost related to work with museum samples highlight the need to consider the limitations of relying on a single barcode.

## Advancements in museomics: Expanding the toolkit for eDNA applications

Museomics—the application of -omics techniques (e.g., genomics, epigenomics) to museum specimens—has opened new possibilities for biodiversity research (Davis and Knapp 2024). Its application resulted in more comprehensive phylogenomic studies (Ruane and Austin 2017, McGuire et al. 2018, Wood et al. 2018, Gauthier et al. 2023), tracking the genetic response of species to recent environmental changes (Bi et al. 2019, Byerly et al. 2022), solving taxonomic uncertainties (Agne et al. 2022b, Muschick et al. 2022, Renner et al. 2024) and more generally reconstructing the evolutionary processes, from population-level to macroevolutionary analyses (Bi et al. 2013, Burrell et al. 2015, Gauthier et al. 2020).

As museomics methods have evolved, the focus has progressively shifted toward genome-wide approaches. Shotgun sequencing of museum specimens has become routine, leading to optimized protocols to retrieve mitochondrial and nuclear genomes, even from highly degraded DNA (Sproul and Maddison 2017, Ferrari et al. 2023, Marsh et al. 2025; see box 1). Beyond providing additional genomic information, shotgun sequencing overcomes the limitations associated with amplicon-based methods when applied to samples with highly degraded DNA or damaged primer binding sites. Building on this progress, long-read sequencing has recently been applied to well-preserved museum specimens, providing enhanced assembly quality and resolution (Bein et al. 2025). However, the fragmented nature of museum specimen DNA often precludes its application, particularly for full nuclear genome reconstruction. In contrast, the reconstruction of complete mitochondrial genomes (mitogenomes) represents a more feasible and valuable target, particularly for improving eDNA reference databases. Mitogenomes are smaller and more abundant in cells, making them easier to recover from degraded material. Computational tools such as MITObim (Hahn et al. 2013) and MitoFinder (Allio et al. 2020) can be valuable for reconstructing sequences, even without a closely related full reference genome. They can facilitate the reconstruction of full mitochondrial genomes even starting from a single barcode sequence, potentially extending the usefulness of museomics for eDNA based studies.

### Beyond barcodes: The role of mitogenomes in eDNA monitoring

Mitogenomes can play a central role in improving the accuracy and resolution of eDNA-based projects. A recurring challenge in the field of eDNA research involves selecting an appropriate barcode that not only encompasses a broad taxonomic range but also offers sufficient resolution for species-level identification. The COI gene—although widely used as a barcode—has been shown to be suboptimal in several taxa, owing to the lack of primer sites that can be used for species-specific amplification (Collins et al. 2019). In addition, the use of a single mitochondrial barcode makes it challenging to reliably distinguish closely related species.

Alternatively, eDNA analysis across multiple gene regions could facilitate detailed ecological assessments and identification of indicator species (Seymour et al. 2020). This multilocus approach has demonstrated the capacity to capture a wide range of organisms and yields reliable taxonomic information (Andres et al. 2021) and the ability to obtain population-level genetic data (Manel et al. 2025). The transition to multilocus approaches is further facilitated by the development of bioinformatic tools (e.g., multilocus database construction and analysis tools; Curd et al. 2019), as well as the availability of multilocus universal primer sets (Wang et al. 2023). Although the specific genetic markers used in future eDNA metabarcoding may vary, sequencing complete mitochondrial genomes could represent an efficient strategy to enable effective metabarcoding in the long term (but see also (Funk and Omland 2003).

Full mitochondrial genomic information can be a valuable resource for reliable species identification (Dziedzic et al. 2023), phylogenetics, and the development of primers for single-species and metabarcoding assays. They are also necessary for transitioning from amplicon-based approaches to capture enrichment (see below and Wilcox et al. 2018) or PCR-free environmental genomics. Mitogenomes reduce issues linked to primer bias (Piñol et al. 2015) and can enable precise quantification of relative species abundance in environmental samples (Yang et al. 2021). In addition, their availability supports the integration of long-read sequencing methods—such as haplotagging—for field-based barcoding and biodiversity surveys (Krehenwinkel et al. 2019). Therefore, recovering mitogenomes from museum specimens could transform eDNA applications, providing that sample contamination issues are effectively addressed.

### Capture-based enrichment: A shared tool for museum and eDNA samples

Contaminations remain a significant concern with museum specimens, as historical DNA can include exogenous material introduced through human handling, microbial colonisation (pre- or post-sampling), or reuse of preservation fluids. Although recent contamination can be minimized during tissue sampling (see box 2), past contamination might lead to sequencing outputs that are biased toward exogenous DNA, due to its higher relative abundance than the degraded endogenous DNA. Although bioinformatic decontamination tools, such as Kraken (Wood and Salzberg 2014), can help identify and remove contamination after sequencing, preventing contamination from being sequenced will yield higher-quality data and reduces the risk of assigning barcodes to the wrong species. Laboratory procedures such as hybrid capture are a solution to enrich endogenous DNA and reduce the amount of exogenous DNA sequenced (Lemmon and Lemmon 2013).

Box 2. **Obtaining tissue samples from natural history collections for molecular analyses.**

Natural history collections increasingly welcome opportunities to use their specimens for scientific research purposes. However, sampling tissues for molecular analyses necessitates careful considerations to prevent contamination and maintain collections integrity. We offer guidance on protocols for obtaining fish tissues from wet collections, but applicable to other taxa preserved in similar conditions. These suggestions should be tailored to project goals, always in consultation with the collection curator.

**Tools and sampling space preparation**

The presence of exogenous DNA from bacteria or fungi or due to human handling is a major concern when working with museum specimens. Although bioinformatic tools such as Kraken (Wood and Salzberg 2014) can help identify and remove contamination after sequencing, preventing contamination at the sampling stage enhances data quality. To limit human-introduced contaminants and cross-contamination, samples must be collected in a sterile environment. The workspace should be thoroughly cleaned using DNA removal solutions or bleach. All tools must be cleaned between samples (e.g., bleach, flame sterilization), and gloves should be changed regularly. Standard dissection equipment and UV-treated 1.5/2-milliliter tubes are recommended. Care must be taken when using bleach and ethanol together, as their combination can generate chloroform.

**Identifying suitable specimens for sampling**

Fish specimens are often formalin fixed, which significantly compromise DNA integrity and impair DNA extraction and library preparation (see box 1). Although protocols are available to retrieve DNA from such specimens (Straube et al. 2021, Hahn et al. 2022), it is preferable to prioritize specimens preserved with alcohol-based fixative to maximize sequencing success (Duval et al. 2010). When preservation history is unknown, visual indicators can help. Bleached eye lenses (white coloration) suggest pure ethanol preservation whereas dark eye lenses often imply formalin fixation (De Bruyn et al. 2011). Specimen collection date also offers clues, since formalin was not used in natural history museums until after 1900 (Simmons 2014). Furthermore, residual formaldehyde concentration can be quantified together with pH, which is often reduced during formalin fixation as formaldehyde breaks down into formic acid (Hahn et al. 2022). Although these indicators can provide hints about preservation, they cannot guarantee the absence of formalin fixation. When feasible, sampling multiple specimens from different lots (e.g., different collection year, collector) can improve sequencing success.

**Selecting the appropriate tissue sample**

The optimal tissue amount for DNA extraction varies among specimens and must balance DNA yield with preserving the integrity of the specimens. An excessive amount of tissue can block the extraction column and may increase PCR inhibitors. Although we generally lack experimental data correlating the amount of tissue to the extracted DNA concentration and quality, extracting several small tissue samples from the same specimen and pooling them can lead to improved results. For fishes, sampling the right flank is ideal, as the left is typically reserved for morphometrics. If the belly is already cut (for formalin fixation), internal organs or muscle can be sampled. Liver often yields higher DNA than skin or muscle (Hahn et al. 2022, Palandačić et al. 2024). If the fish specimen is intact, gill filaments can be sampled. Alternatively, a biopsy punch or small incision allow to retrieve tissue from beneath the skin. For particularly small or valuable specimens, needlepoint nondestructive internal tissue sampling may be used (Haÿ et al. 2020).

Following specimen collection, tissue should be stored in 95% ethanol. Ethanol with a lower concentration contains more water, which may hinder preservation. To mitigate ethanol evaporation, screw-top tubes with a rubber seal are preferred, in addition to long-term storage at –80 degrees Celsius when possible (the colder the better). Further evaporation prevention can be achieved by sealing the tube with parafilm.

Capture protocols involve creating molecular probes complementary to the target region of interest to capture desired genomic regions while minimizing exogenous DNA contamination. Hybrid capture protocols can target a range of regions of interest in the genome, from single barcode (see the “Barcoding of historical museum specimens for eDNA reference databases” section) to mitogenomes (Evans et al. 2019), ultraconserved elements (Derkarabetian et al. 2019), universal single-copy orthologs (Dietz et al. 2024), or curated population genomic markers containing informative single-nucleotide polymorphisms (e.g., Manel et al. 2025). While benchtop protocols such as long-range PCR (Bekaert et al. 2016; González Fortes and Paijmans 2019) or restriction-site–associated DNA–derived methods like HyRAD (Suchan et al. 2016) and HyRAD-X (Schmid et al. 2017) enable the production of custom probes, fully synthesised probes for in-solution hybridisation capture can now be obtained through commercial synthesis platforms and kits. By selectively capturing and amplifying specific genomic targets, these approaches enhance the efficiency of sequencing DNA from museum samples. The probes required are still expensive to produce, but their costs might decrease in the future, notably thanks to the invention of benchtop DNA printers (Grinstein 2023).

Because both museum specimens and eDNA samples involve degraded DNA and complex mixtures of target and non-target DNA, innovations in capture-based techniques developed for historical samples are highly relevant to eDNA-based biodiversity assessments. The field of eDNA capture is relatively new and holds great potential for improving the efficiency and accuracy of metabarcoding studies (Manel et al. 2025). Ultimately, it could also potentially result in a PCR-free eDNA approach, resolving the issues associated with PCR amplification biases (Piñol et al. 2015). However, unlike most museomics research, eDNA studies target a wide spectrum of species that may exhibit high divergence. This raises the question of which probe sequences to use. One strategy involves using barcode gene DNA from a distantly related species that is approximately equidistant to all the studied species (Mariac et al. 2018). Another approach involves aggregating available DNA sequences from databases and employing *in silico* approaches to generate a set of probes that summarizes the information within those sequences, a method successfully applied in various museomics studies (e.g., Li et al. 2023, Dietz et al. 2024, Geburzi et al. 2024). By integrating the advantages of capture-based methods with the extensive resources of natural history museum collections, we could further address the taxonomic and geographic gaps in eDNA reference databases.

## Building comprehensive and carefully curated mitogenome databases

In order to circumvent limitations of approaches related to single barcode use (see above), a promising approach could be to shift the focus toward the development of extensive and curated mitogenomic databases. With repositories containing vouchered samples and comprehensive mitogenomic data, researchers can access the necessary genetic information for the effective use of eDNA in biodiversity studies (de Santana et al. 2021). Efforts to improve mitogenome databases are ongoing (e.g., Zhu et al. 2023), and guidelines are available to create regional databases of mitogenome sequences for target taxa (Dziedzic et al. 2023). Integrating specimen metadata and morphological data with mitogenome sequences in the database advances scientific collection digitisation and aligns with the extended specimens concept (Chang 2020) and the emerging field of collectomics (Sigwart et al. 2025). An extended specimen comprises both the physical specimen and associated data, including measurements, environmental information, photographs or X-rays, DNA sequences, and parasites or symbionts found on the material. Explicitly linking the specimen to other data—including DNA sequences and subsequent analysis incorporating the specimen's information—could help define the level of confidence we can have in a given sequence used for eDNA-based research.

## Considerations regarding the use of museum specimens

Although museum specimens can serve as valuable resources for enhancing eDNA reference databases, certain limitations exist. One of the key limitations is related to reliable taxonomic identification. When working with historical collections, the taxonomic classifications can be inaccurate, since species definitions and concepts have evolved, and species identification provided by the museum may not always be done by expert taxonomists. To overcome sequencing misidentified specimens, the optimal approach would be to only sample holotypes or paratypes, even if accessing these can be challenging. Indeed, type specimens are valuable to natural history museum collections and may not be readily available for DNA sampling if the integrity of morphological characters is jeopardized. A practical alternative is to ask qualified taxonomists to verify the identity of candidate specimens before sequencing, ensuring reliable reference data (Engel et al. 2021). In addition to taxonomic uncertainty, sampling locations and dates might be incomplete or unreliable. In consequence, although specimen labels provide the primary layer of scientific information, they are not infallible: Misidentifications, transcription mistakes, and even deliberate falsifications have all been documented (Phillips et al. 2019). Label information on the specimen should therefore be verified independently—through expert reexamination, georeferencing checks, or molecular confirmation—before being used as a genetic reference (Boessenkool et al. 2010, Rawlence et al. 2014, Palandačić et al. 2020)

An additional limitation is the scarcity of data on the curatorial handling of museum specimens, which is helpful for the application of museomics to wet collections. Information on critical parameters such as formalin concentration, duration of formalin exposure, pH levels, refilling practices, and storage temperatures could help better understand and anticipate the potential degradation of DNA within these specimens, but this data is infrequently recorded. This also raises concerns about the potential contamination of museum specimens, arising from handling routines or from bacterial and fungal growth over time.

### Ethical challenges related to specimens’ origins

It is important to acknowledge that many of the specimens in natural history museum collections were collected in locations outside the museum's country of origin. To address this, we should explore ways to meaningfully engage researchers from the countries where these specimens were sampled. This could involve collaborating with taxonomists and biodiversity experts from the countries of origin to validate the identifications of taxa and facilitate their participation in the research process. Making the data and findings from museum-based studies openly accessible online would also enable equitable recognition and participation of researchers from the countries of specimen origin. Natural history museums have invested significant resources to digitize their collections and make them widely accessible through online platforms such as the Global Biodiversity Information Facility (GBIF). These collaborative data-sharing initiatives represent important steps toward a more inclusive approach to leveraging museum collections for research.

## Conclusions

Natural history collections are inherently interdisciplinary, linking various fields such as taxonomy, genetics, and conservation. Through both their collections and their role as scientific institutions, natural history museums have the potential to contribute to the advancement of eDNA research for biodiversity monitoring. They offer a vast resource of museum specimens that remain underused in genetic research and also have the potential to serve as repositories for freshly collected eDNA samples (de Santana et al. 2021). Many species without available DNA barcodes can be found in these collections, providing an opportunity to fill gaps in reference databases. In addition, the methodological advances developed for working with fragmented DNA from museum specimens are often directly transferable to the analysis of eDNA. Both sources frequently involve short and degraded DNA sequences. When appropriate genomic regions are targeted and sequenced, they can provide useful taxonomic information. Well-developed museomics methodologies, such as hybrid capture, could potentially be applied to eDNA to improve the recovery and sequencing of target taxa. Ultimately, the integration of museomics into eDNA research has the potential to enhance our understanding of global biodiversity, facilitate effective environmental monitoring, and support conservation efforts.

## Supplementary Material

biaf140_Supplemental_Files

## Notes

Sarah Schmid (sarah.schmid@ik.me) is a postdoctoral research assistant, Camille Albouy is a lead scientist, and Loïc Pellissier is a group leader at the Ecosystems and Landscape Evolution group in between the Swiss Federal Institute for Forest, Snow and Landscape Research in Birmensdorf, Switzerland and ETH Zürich, in Zürich, Switzerland. Nicolas Straube is the curator of the ichthyology collection at the University Museum of Bergen, in Bergen, Norway. Bo Delling is the curator of the fish collection at the Swedish Museum of Natural History, in Stockholm, Sweden. James Maclaine is a curator of the fish collection at the Natural History Museum, in London, England, in the United Kingdom. Michael Matschiner is the director of the Bavarian State Collection of Zoology in Munich, Germany and a professor at the Ludwig Maximilians University Munich, in Munich Planneg, Germany, and is also affiliated with the Natural History Museum at the University of Oslo, in Oslo, Norway. Peter Rask Møller is the curator of the ichthyology collection at the Natural History Museum of Denmark, in Copenhagen, Denmark. Annamaria Nocita is the curator of the fish collection in the museum La Specola, in Florence, Italy. Anja Palandačić is the collection manager of the fish collection in the Museum of Natural History Vienna, in Vienna, Austria, and is also affiliated with the Department of Biology at the University of Ljubljana, in Ljubljana, Slovenia. Lukas Rüber is the curator of the ichthyology collection at the Natural History Museum of Bern, in Bern, Switzerland and is also affiliated with the Institute of Ecology and Evolution at the University of Bern, in Bern, Switzerland. Moritz Sonnewald is the curator of the fish collection at the Senckenberg Research Institute in Frankfurt am Main, Germany. Nadir Alvarez is the director of the Naturéum—State Museum of Natural History of the Canton de Vaud, in Lausanne, Switzerland and is also affiliated with the Department of Ecology and Evolution at the University of Lausanne, in Lausanne, Switzerland. Stéphanie Manel is a professor at the Ecole Pratique des Hautes Etudes and is based at the Centre d'Ecologie Fonctionnelle et Evolutive, in Montpellier, France, and is a member of the Institut Universitaire de France, in Paris, France.

## Notes

The scripts to generate the data sets and plots are available on Dryad at https://doi.org/10.5061/dryad.0zpc8677g.
