# Supplementary material for: Unlocking natural history collections to improve eDNA reference databases and biodiversity monitoring
Source: Bioscience. 2025 Sep 11;75(12):1083–95. doi: 10.1093/biosci/biaf140 (PMC12683532; doi:10.1093/biosci/biaf140)
Supplement: biaf140_Supplemental_Files [file biaf140_supplemental_files.zip › supp_figures_mm.docx]

# Supplemental figures and material and methods for “Combining eDNA and museomics to enhance biodiversity monitoring”

Sarah Schmid, Nicolas Straube, Camille Albouy, Bo Delling, James Maclaine, Michael Matschiner, Peter Rask Møller, Annamaria Nocita, Anja Palandačić, Lukas Rüber, Moritz Sonnewald, Nadir Alvarez, Stéphanie Manel, Loïc Pellissier

## Content

[Figure S1](#_Figure_S1). Potential of museum specimens to improve reference databases according to geographic regions.

[Figure S2](#_Figure_S2). Potential of museum specimens to improve reference databases according to global IUCN Red List of Threatened Species assessments.

[Supplemental material and method](#_Supplemental_material_and)

[References](#_References)

Supplemental tables are in a separate excel file (supp_tables.xlsx)

## Supplemental figures

### Figure S1


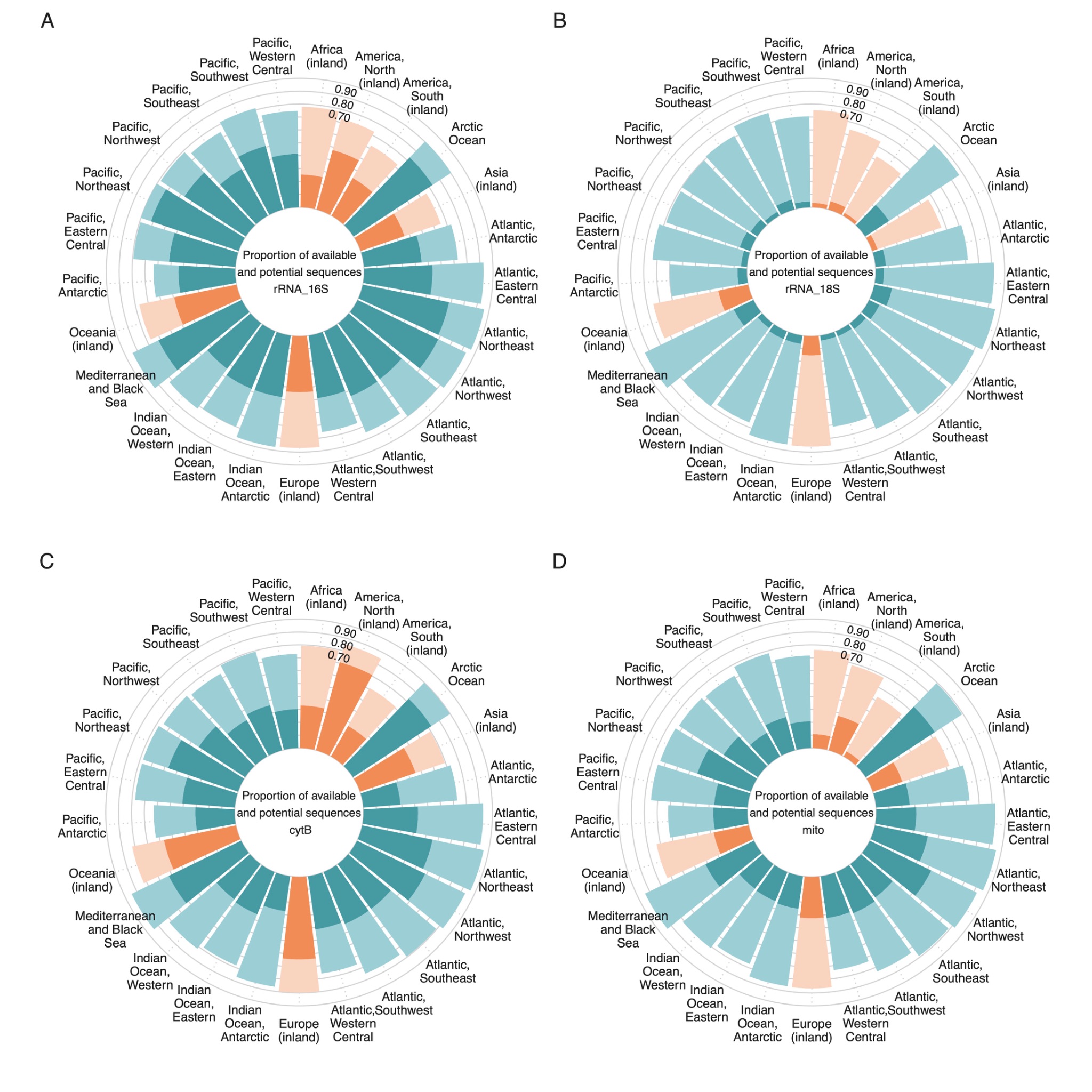


Figure S1. Potential of museum specimens to improve reference databases for 16S ribosomal RNA (16S) (A), 18S ribosomal RNA (18S) (B) and cytochrome B (cytB) (C) mitochondrial genes used in eDNA studies and for the full mitochondrial genome (D) according to geographic regions. Regions in blue are hosting marine species while regions in orange are for freshwater species (inland regions). Proportion of sequences already available is highlighted in dark colour. Potential new sequences based on available museum specimens in European collections are displayed in lighter colour. Potential of improvement according to geographic regions. The complete list of fish species was retrieved from FishBase using the R package rfishbase (Boettiger et al. 2012) as well as their corresponding geographic distribution. The museum data were retrieved from GBIF.

### Figure S2


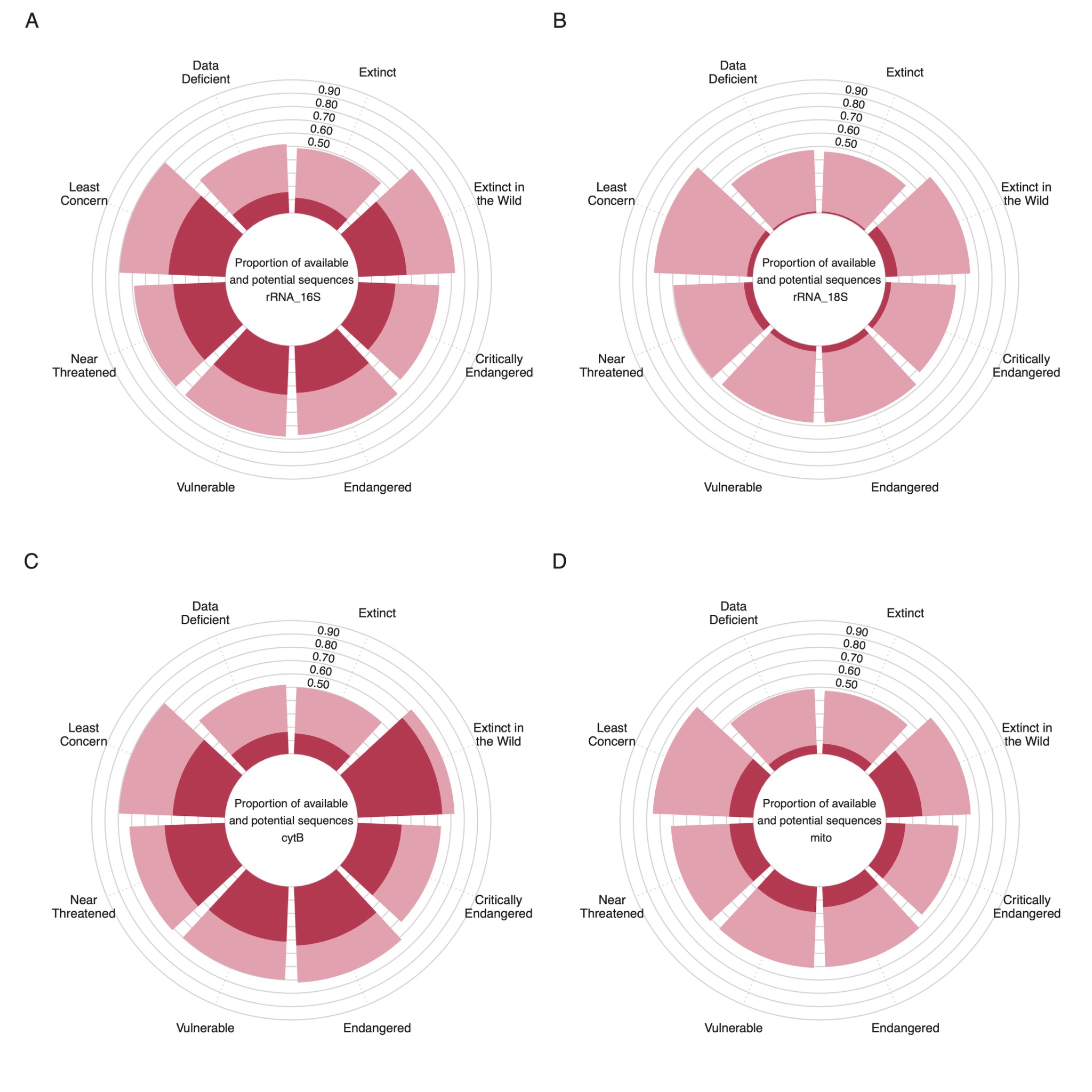
Figure S2. Potential of museum specimens to improve reference databases for for 16S ribosomal RNA (16S) (A), 18S ribosomal RNA (18S) (B) and cytochrome B (cytB) (C)mitochondrial genes used in eDNA studies and for the full mitochondrial genome (D) according to global IUCN Red List of Threatened Species assessments. Proportion of sequences already available is highlighted in dark colour. Potential new sequences based on available museum specimens in European collections are displayed in lighter colour. The complete list of fish species was retrieved from FishBase using the R package rfishbase (Boettiger et al. 2012). The museum data were retrieved from GBIF. The IUCN Red List assessments were retrieved from the IUCN website.

## Supplemental material and methods

### Data retrieval and comparison of eDNA barcodes sequences with museum specimen records

To examine the cumulative number of species sequenced for a given DNA barcode/mitochondrial genome (also referred as mitogenome) over the years, we retrieved all data available from NCBI using the R package rentrez v1.2.3 (Winter 2017). We searched the nucleotide database for the rRNA 12S, rRNA 16S, rRNA 18S, cytochrome B (cytB), cytochrome oxydase I (COI) barcodes as well as for the complete mitogenomes for all fish orders. In addition, we also retrieved all the fish species with available data on the sequence read archive (SRA) using the Entrez Direct (Kans 2024), which provides access to the NCBI databases from a Unix terminal window.

To highlight the potential of museum specimens for increasing the number of species with an available barcode/mitogenome sequence, we first downloaded all available datasets on the Global Biodiversity Information Facility (GBIF) listing fish specimens stored in European natural history museum collection (see table S1). Subsequently, we downloaded a list of all existing fish species using the R package rfishbase v5.0.0 (Boettiger et al. 2012) and extracted their geographic range (field AreaCode). In addition, we retrieved information about the Red List status of all fish species from the International Union for Conservation of Nature and Natural Resources (IUCN) website. All the datasets (barcodes, museum specimens, IUCN status and fish species list, and geographic range) were combined in R v.4.3.0 and subsequently plotted. All the scripts to retrieve data and to generate the figures are available on Dryad (DOI: 10.5061/dryad.0zpc8677g).

## References

Boettiger C, Lang DT, Wainwright PC. 2012. rfishbase: exploring, manipulating and visualizing FishBase data from R. *Journal of Fish Biology* 81: 2030–2039.

Kans J. 2024. *Entrez direct: E-utilities on the UNIX command line*. In Entrez programming utilities help [Internet]. National Center for Biotechnology Information (US).

Winter DJ. 2017. rentrez: An R package for the NCBI eUtils API. *The R Journal* 9: 520–526.
